# New Insights into *Dehalococcoides mccartyi* Metabolism from a Reconstructed Metabolic Network-Based Systems-Level Analysis of *D. mccartyi* Transcriptomes

**DOI:** 10.1371/journal.pone.0094808

**Published:** 2014-04-14

**Authors:** M. Ahsanul Islam, Alison S. Waller, Laura A. Hug, Nicholas J. Provart, Elizabeth A. Edwards, Radhakrishnan Mahadevan

**Affiliations:** 1 Department of Chemical Engineering and Applied Chemistry, University of Toronto, Toronto, Ontario, Canada; 2 Department of Cell and Systems Biology, University of Toronto, Toronto, Ontario, Canada; 3 Institute of Biomaterials and Biomedical Engineering, University of Toronto, Toronto, Ontario, Canada; 4 European Molecular Biology Laboratory (EMBL), Heidelberg, Germany; 5 Department of Earth and Planetary Science, University of California, Berkeley, California, United States of America; University of North Carolina at Charlotte, United States of America

## Abstract

Organohalide respiration, mediated by *Dehalococcoides mccartyi*, is a useful bioremediation process that transforms ground water pollutants and known human carcinogens such as trichloroethene and vinyl chloride into benign ethenes. Successful application of this process depends on the fundamental understanding of the respiration and metabolism of *D. mccartyi.* Reductive dehalogenases, encoded by *rdh*A genes of these anaerobic bacteria, exclusively catalyze organohalide respiration and drive metabolism. To better elucidate *D. mccartyi* metabolism and physiology, we analyzed available transcriptomic data for a pure isolate (*Dehalococcoides mccartyi* strain 195) and a mixed microbial consortium (KB-1) using the previously developed pan-genome-scale reconstructed metabolic network of *D. mccartyi*. The transcriptomic data, together with available proteomic data helped confirm transcription and expression of the majority genes in *D. mccartyi* genomes. A composite genome of two highly similar *D. mccartyi* strains (KB-1 *Dhc*) from the KB-1 metagenome sequence was constructed, and operon prediction was conducted for this composite genome and other single genomes. This operon analysis, together with the quality threshold clustering analysis of transcriptomic data helped generate experimentally testable hypotheses regarding the function of a number of hypothetical proteins and the poorly understood mechanism of energy conservation in *D. mccartyi*. We also identified functionally enriched important clusters (13 for strain 195 and 11 for KB-1 *Dhc*) of co-expressed metabolic genes using information from the reconstructed metabolic network. This analysis highlighted some metabolic genes and processes, including lipid metabolism, energy metabolism, and transport that potentially play important roles in organohalide respiration. Overall, this study shows the importance of an organism's metabolic reconstruction in analyzing various “omics” data to obtain improved understanding of the metabolism and physiology of the organism.

## Introduction

Obligate anaerobes such as *Dehalococcoides mccartyi* support growth and metabolism by conserving energy from an unusual respiratory metabolic process termed organohalide respiration [1]–[3]. The hallmark of this important biological process lies in the detoxification of halogenated xenobiotics such as trichloroethene and vinyl chloride — known human carcinogens and groundwater pollutants — as well as tetrachloroethene, chlorobenzenes, dioxins, and polychlorinated biphenyls [4]–[7]. However, optimized use of this natural and effective bioremediation process is hampered due to the lack of detailed knowledge about *D. mccartyi* metabolism, both in pure cultures and in mixed microbial communities they normally inhabit. Although some of the genes and enzymes involved in organohalide respiration are identified and characterized [8]–[11], mechanism of the respiratory chain and its components, as well as functional annotations of ∼50% *D. mccartyi* genes is yet to be determined [12], [13]. Due to the associated difficulty in expressing genes heterologously and the lack of a genetic system in *D. mccartyi* [14], experimental studies on characterization and manipulation of genes and enzymes of these organisms are challenging. Hence, most studies to date have primarily focused on the identification and characterization of reductive dehalogenase homologous (*rdh*) genes, and their respective enzyme's cofactors and substrate ranges [8], [10], [15]–[18].

Recently, a number of isotope labeling studies concerning *D. mccartyi* metabolism have discussed the genes and enzymes of some key metabolic processes, including the TCA-cycle, and amino acid transport and metabolism [19]–[21]. In addition, sequencing of multiple *D. mccartyi* genomes [12], [13], [22] enabled the construction of a detailed pan-genome-scale constraint-based model of metabolism, which revealed their energy-starved nature, as well as depicted the overall metabolic landscape of *D. mccartyi* [23]. Also, a number of proteomic studies [24]–[26] have provided important information on some metabolic genes and processes, including nitrogen fixation and carbon metabolism of *D. mccartyi*. Apart from these metabolic studies, data from systems-wide high-throughput experimental studies such as whole genome microarrays are available for *D. mccartyi* strain 195 (formerly, *Dehalococcoides ethenogenes* strain 195) [27]–[29]. A shotgun metagenome microarray study on KB-1 — a *D. mccartyi*-containing dechlorinating mixed microbial community — has been published recently [30], [31]. While these studies obtained expression data for all genes, each study focused on analyzing the expression of specific genes involved in, for instance, reductive dechlorination and energy conservation, in cobalamin (vitamin B12) biosynthesis pathway, or phage related genes. None of these studies focused on the analysis of overall *D. mccartyi* metabolism using genome-wide transcriptomic data. Also, no integrated analysis of the available transcriptomic and proteomic data with the pan-genome-scale metabolic network of these bacteria [23] has been conducted yet. Such a systemic analysis of “omics” data can be useful to glean a more comprehensive understanding of the unusual metabolism of *D. mccartyi*, as well as to verify the presence of sequenced genes in their genomes as most genes have only weak bioinformatic evidence.

Here, we analyzed the published transcriptomic data for a pure culture, *Dehalococcoides mccartyi* strain 195 (from here on, strain 195) [27], [28] and a mixed culture, KB-1 [30], [31] using the previously developed pan-genome-scale *D. mccartyi* metabolic network [23] as a guide. A composite genome of two highly similar *D. mccartyi* strains in KB-1 (from here on, KB-1 *Dhc*) was constructed from the publicly available KB-1 metagenome sequences (genome.jgi-psf.org/aqukb/aqukb.download.ftp.html) and subsequently used for analyzing *D. mccartyi*-specific transcriptomic data from the KB-1 community arrays [30], [31]. This metabolic network-guided study of transcriptomic data, together with available proteomic data analyzed and confirmed the transcription and expression of the majority genes in strain 195 and KB-1 *Dhc* genomes. In addition, we specifically examined and visualized the expression of some metabolic genes and hypothetical proteins, as well as their putative annotations proposed during the metabolic modeling study [23]. Then, operon analysis for the KB-1 *Dhc* genome and other single strain-genomes of *D. mccartyi*, including strains 195, CBDB1, and GT was conducted. The transcriptomic data were further analyzed with the quality threshold (QT) clustering algorithm and functional enrichment analysis, which provided interesting insight on the poorly understood mechanism of energy conservation in these bacteria. Moreover, these bioinformatic analyses of transcriptomic data, along with operon analysis helped suggest putative functions for at least five hypothetical proteins of strain 195. Thus, our metabolic reconstruction-based meta-analysis provides a guide for selecting and screening some of the hypothetical proteins in *D. mccartyi* genomes, which can aid future targeted proteomic work to increase our knowledge on the physiology and biochemistry of these useful bacteria.

## Results and Discussion

### Analyzing the differences between strain 195 and KB-1 *Dhc* transcriptomic data with principal component analysis

**Figure 1:**
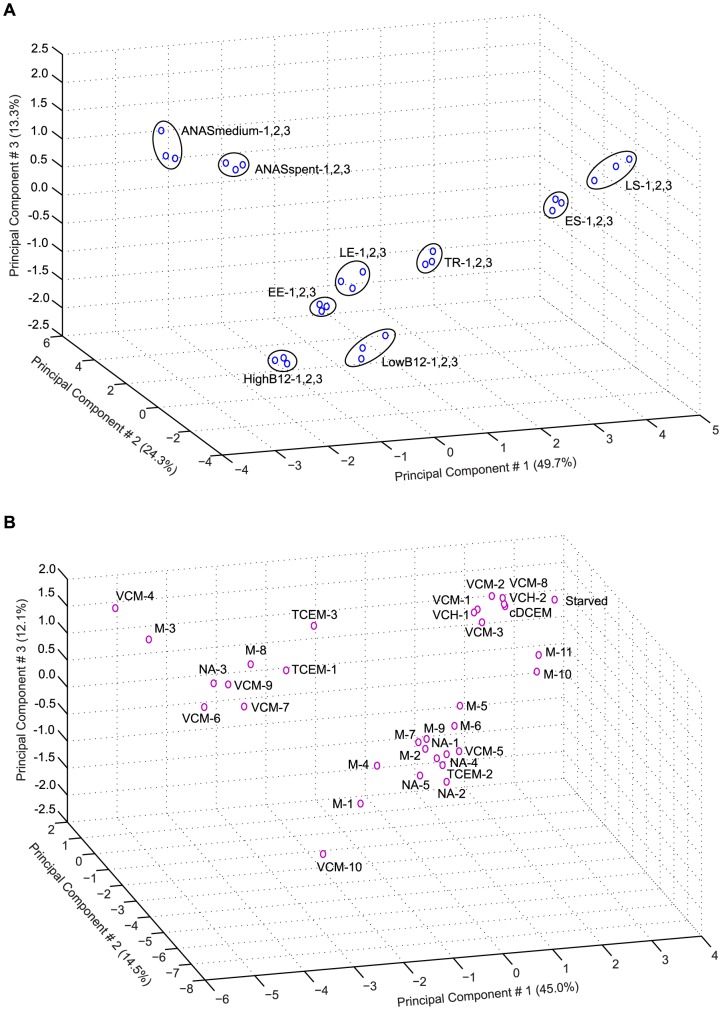
Principal component analysis (PCA) of array data for strain 195 and KB-1 *Dhc* samples. (A) Array data for pure culture strain 195 included triplicate biological replicates that were clustered together for each experimental condition by PCA. All samples were used for subsequent data analysis. (B) *D. mccartyi*-specific array data for biological replicates of KB-1 mixed culture demonstrated variability owing to array type, experimental design, and complex interactions of organisms in the community. Subsequent data analyses, therefore, were conducted with the expression values of all 33 biological replicates. “EE”  =  early exponential phase, “LE” =  late exponential phase, “TR”  =  transition phase, “ES”  =  early stationary phase, “LS”  =  late stationary phase, “HighB12”  =  higher concentration of vitamin B12 in the medium, “LowB12”  =  lower concentration of vitamin B12 in the medium, “ANASspent”  =  ANAS supernatant added medium, “ANASmedium”  =  growth medium of ANAS cultures, “TCEM”  =  trichloroethene and methanol, “cDCEM”  =  cis 1,2-dichloroethene and methanol, “VCM”  =  vinyl chloride and methanol, “VCH”  =  vinyl chloride and hydrogen, “M”  =  methanol only, “NA”  =  not amended.

Principal component analysis (PCA) is a useful statistical method to identify underlying trends of a high-dimensional data set such as transcriptomic data from microarray experiments by reducing its dimensionality and extracting important information [32]–[34]. PCA was performed for strain 195 and KB-1 *Dhc* array data to analyze their dimensionality and variability (Figure 1). In total, published data from 27 strain 195 samples under 9 conditions (Figure 1A) and 33 KB-1 *Dhc* samples under 7 conditions were analyzed by PCA (Figure 1B) [27], [28], [30], [31]. Strain 195 samples (Figure 1A) were collected from parallel triplicate cultures during sequential dechlorination of trichloroethene (TCE) at 5 time points: Early Exponential (EE), Late Exponential (LE), Transition (TR), Early Stationary (ES), and Late Stationary (LS), in high and low vitamin B12 concentrations (HighB12 and LowB12), and in two different growth media with higher nutrient contents (ANASmedium and ANASspent) [27], [28]. ANAS is an enrichment culture of a *D. mccartyi*-containing methanogenic mixed microbial community [35], [36], and array experiments were conducted with strain 195 in the ANAS mineral medium (ANASmedium), as well as in the filter sterilized supernatant of the ANAS culture (ANASspent) [28]. The PCA-plot (Figure 1A) shows good agreement between triplicate samples for the corresponding conditions, indicating that the biological replicates behaved consistently in the array experiments.

The samples used for extracting RNA to interrogate KB-1 *Dhc* arrays were comparisons of mainly two growth conditions: one with and one without a chlorinated electron acceptor [30], [31]; specifically, KB-1 cultures grown with trichloroethene and methanol (TCEM) were compared to cultures grown with methanol (M) only. Other conditions tested included cis-1,2-dichloroethene and methanol (cDCEM), vinyl chloride and methanol (VCM), and vinyl chloride and hydrogen (VCH). These samples were also compared to samples that were not amended with any substrates for 4 days (NA) and for 1 year (“Starved”) (Figure 1B). Although methanol is supplied to KB-1 as the electron donor, it is fermented to H_2_ which is the direct electron donor for *D. mccartyi* strains in KB-1 [31], [37]. RNA for the cDCEM and starved conditions was arrayed only once while multiple biological replicates for other conditions were analyzed (TCEM: 3 samples, VCM: 10 samples, VCH: 2 samples, M: 11 samples, and NA: 5 samples). PCA showed high dimensionality in KB-1 *Dhc* array data (Figure 1B), which primarily stemmed from the type of array technology (shotgun “spotted” DNA array) and the experimental approach used (sample collection for only one time point 4 hours after substrate addition), as well as the inherent variability of working with a mixed microbial culture.

### Improved identification and confirmation of *D. mccartyi* genes with transcriptomic and proteomic data

Of the total 1560 putative genes in strain 195 genome [13], only 3 were experimentally characterized: DET0079 (*tce*A) [17], DET0318 (*pce*A) [18], and DET1363 (*mgs*D) [38]. However, the 1162 putative genes of KB-1 *Dhc* draft genome have only bioinformatic evidence. Due to the lack of biochemical evidence for the majority genes in *D. mccartyi* genomes, available high-throughput experimental data such as proteomic [24], [25], [39] and transcriptomic [27], [28], [30] data can be used to identify and support the existence of these putative genes, if not their functions. Previous proteomic studies [24], [25], [39] identified only 718 strain 195 and 106 KB-1 *Dhc* genes (Tables S1 and S2 in File S1). However, the transcriptomic data for both organisms, analyzed in this study, showed that 925 strain 195 genes and 257 KB-1 *Dhc* genes were transcribed or “on” (see Materials and methods for how gene transcription cut-off values were chosen to determine “on” and “off”) in all samples. Among these genes, 624 and 19 of strain 195 and KB-1 *Dhc*, having proteomic evidence, were actually expressed in all samples (Tables S1 and S2 in File S1). In addition, only 229 and 34 genes from strain 195 and KB-1 *Dhc* were found to be “off” or not transcribed in all samples, and the remaining genes (406 strain 195 and 871 KB-1 *Dhc*) were transcribed in at least one sample (Tables S1 and S2 in File S1). Thus, the majority (∼60%) of strain 195 genes were transcribed in all samples, while the majority (∼75%) of KB-1 *Dhc* genes were transcribed in some samples but not all. Further analysis of the proteomic and transcriptomic evidence for hypothetical proteins and metabolic genes were discussed in the following sections.

### Confirming the expression of *D. mccartyi* hypothetical proteins from transcriptomic and proteomic data

Hypothetical proteins and genes with unknown functions constitute ∼33% (523) of strain 195 and ∼22% of KB-1 *Dhc* (264) genomes, the latter being a draft genome. Analysis of transcriptomic and proteomic data for these genes revealed transcription of 243 strain 195 (Table 1 and Table S3 in File S1) and 56 KB-1 *Dhc* (Table 1 and Table S5 in File S1) hypothetical proteins in all samples. Due to having proteomic evidence, 96 and 1 of these hypothetical proteins of strain 195 and KB-1 *Dhc* were (Table 1 and Tables S3 and S5 in File S1) actually expressed. The majority of KB-1 *Dhc* hypothetical proteins (208), including 4 with proteomic evidence (Table 1 and Table S6 in File S1), were transcribed or “on” in some samples but not all, while strain 195 had 164 such genes (Table 1 and Table S4 in File S1). Thus, 78% of strain 195 and 98% of KB-1 *Dhc* hypothetical proteins were transcribed in at least one sample, and this result is relatively high in comparison to 33% and 30% expressed hypothetical proteins in *Shewanella oneidensis* [40] and *Geobacter sulfurreducens* [41], identified in similar transcriptomic studies. Among all hypothetical proteins, only 116 strain 195 (Table S4 in File S1) and 6 KB-1 *Dhc* (Table S6 in File S1) were found to be “off” in all samples, and none of these genes was identified in previous proteomic studies.

**Table 1:** Transcriptomic and proteomic evidence available for hypothetical proteins and metabolic genes of strain 195 and KB-1 *Dhc*

Category	Strain 195	KB-1 *Dhc*
**Data for hypothetical proteins**		
Highly transcribed in all samples without proteomic evidence	147	55
Highly transcribed in all samples with proteomic evidence (i.e., expressed)	96	1
Highly transcribed in some samples without proteomic evidence	150	198
Highly transcribed in some samples with proteomic evidence (i.e., expressed)	14	4
Not transcribed in any sample (“off”)	116	6
**Data for metabolic genes**		
Highly transcribed in all samples without proteomic evidence	48	85
Highly transcribed in all samples with proteomic evidence (i.e., expressed)	266	16
Highly transcribed in some samples without proteomic evidence	59	269
Highly transcribed in some samples with proteomic evidence (i.e., expressed)	34	48
Not transcribed in any sample (“off”)	60	11

### Confirming the expression of *D. mccartyi* metabolic genes from transcriptomic and proteomic data

**Figure 2:**
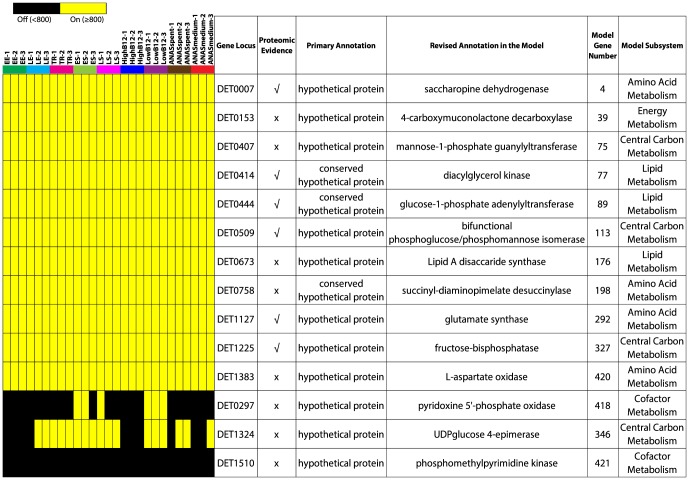
Proteomic and transcriptomic evidence for the hypothetical proteins of strain 195 reannotated in the *D. mccartyi* metabolic model. Transcriptomic evidence for the reannotated hypothetical proteins is presented as heat maps while proteomic evidence is obtained from literature [24], [25]. Proposed functions and the metabolic pathways in which the hypothetical proteins were involved in the metabolic model are also shown in the table.

**Figure 3:**
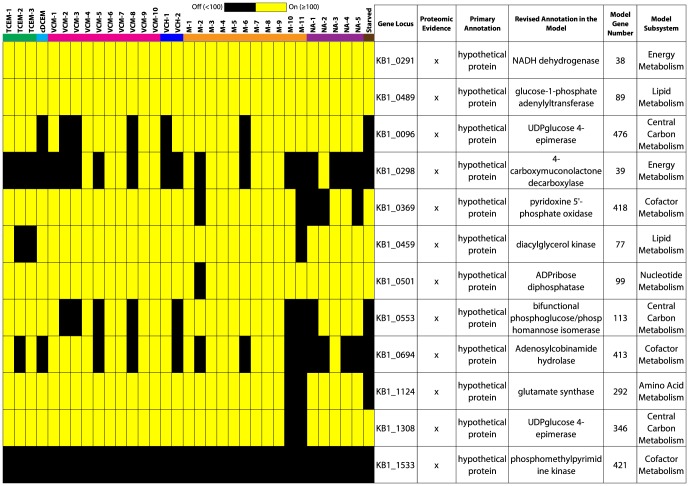
Proteomic and transcriptomic evidence for the hypothetical proteins of KB-1 *Dhc* reannotated in the *D. mccartyi* metabolic model. Transcriptomic evidence for the reannotated hypothetical proteins is presented as heat maps while proteomic evidence is obtained from literature [25], [39]. Proposed functions and the metabolic pathways in which the hypothetical proteins were involved in the metabolic model are also shown in the table.

Metabolic genes from the transcriptomic data were identified by mapping them to the manually curated pan-genome-scale metabolic model for *D. mccartyi* [23] (see Materials and methods, and Figures S1 and S2 in File S2). This analysis led to the identification of 467 and 429 metabolic genes for strain 195 and KB-1 *Dhc*, respectively (Tables S7 and S8 in File S1). Of the 467 putative metabolic genes, 314 were transcribed or “on” in all strain 195 samples, 93 were “on” in at least one sample, and 60 were “off” or not transcribed in any sample (Table 1). Also, the majority (58% or 300) of these metabolic genes were detected in previous proteomic studies (Table 1 and Table S7 in File S1) [24], [25] and hence considered expressed. In contrast, only 64 metabolic genes of KB-1 *Dhc*, having proteomic evidence, were actually expressed (Table S8 in File S1) [25], [39]. Nonetheless, 101 KB-1 *Dhc* metabolic genes were transcribed in all samples, 317 were “on” in at least one sample, and only 11 were “off” in all samples (Table S8 in File S1). In total, the presence of 407 strain 195 and 418 KB-1 *Dhc* metabolic genes were supported by transcriptomic data. Most importantly, 13 strain 195 (Figure 2) and 11 KB-1 *Dhc* (Figure 3) metabolic genes, which were originally annotated as hypothetical proteins and reannotated during the metabolic modeling study [23], were transcribed in at least one sample. Being detected in proteomic or transcriptomic studies, these hypothetical proteins are good candidates for future biochemical experiments to prove their proposed gene functions.

**Figure 4:**
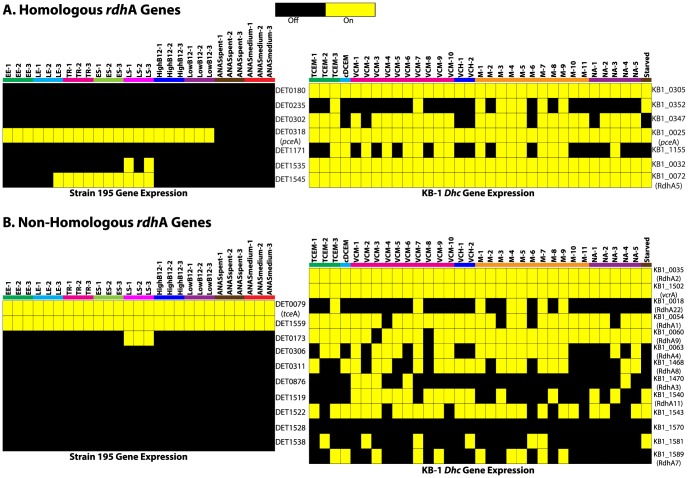
Expression of reductive dehalogenase homologous (*rdh*A) genes. Absolute intensities of (A) homologous and (B) non-homologous *rdh*A genes of strain 195 and KB-1 *Dhc* are illustrated as heat maps. For strain 195 data, the characterized genes, *tce*A and *pce*A [17], [18], and DET1559 were highly expressed as previously reported [42], [43]. DET1545 and its homolog in KB-1 *Dhc*, KB1_0072, were expressed at highest levels in late stationary or unamended conditions (to see this more clearly, refer to absolute values of intensities provided in Tables S9 and S10 in File S1). For KB-1 *Dhc rdh*A genes, identifiers in parenthesis are provided for cross-referencing as they were used in other studies [26], [31], [42]. Although *vcr*A and *pce*A homologs were found, *bvc*A and *tce*A homologs were not identified as probes in the KB-1 *Dhc* shotgun arrays. Note that 12 out of 20 *rdh*As from KB-1 *Dhc* were found to be “on” even in the “Starved” condition.

Further analysis of the transcriptomic data for metabolic genes identified the presence of more *rdh*A genes — involved in the energy conserving reductive dechlorination reaction — for KB-1 *Dhc* (20 *rdh*As) than for strain 195 (17 *rdh*As) (Figure 4, and Tables S9 and S10 in File S1), and 7 of those were homologous to strain 195 *rdh*A genes (Figure 4A). Among the strain 195 *rdh*A genes, only 2 (DET1559 and DET0079, *tce*A) out of 17 were transcribed in all samples (Figures 4A and 4B); *tce*A was transcribed because TCE was used as the electron acceptor in all samples, but the expression of DET1559 seemed to be constitutive as noted previously [8], [25], [26]. Also notable is DET1545 which, similar to previous studies [42], [43], was transcribed even in the stationary phase when the substrate concentration was low (Figure 4A). The KB-1 *rdh*A genes included homologs of the characterized *pce*A [18] and *vcr*A genes [10], [31], [44]; however, probes for homologs of other characterized *rdh*As (*bvc*A [15] and *tce*A [17]) were not present in KB-1 *Dhc* shotgun arrays. A recent proteomic study [39] of KB-1 identified 5 *rdh*As, including *vcr*A (KB1_1502), *bvc*A (KB1_6), *tce*A (KB1_1037), RdhA5 (KB1_0072), and RdhA1 (KB1_0054). In total, 6 out of 17 KB-1 *rdh*As were transcribed in all samples, while only one *rdh*A gene (KB1_1570) was “off” in all samples (Figures 4A and 4B). Most importantly, a total of 12 KB-1 *rdh*As were transcribed even in the starved condition (Figures 4A and 4B), indicating that the genes were not strictly regulated to the presence of chlorinated substrates. This notion is further evident from the *rdh*A expression profiles (Figures 4A and 4B), which do not show any major difference between the samples with chlorinated solvents and those without. All the M and NA samples showed almost similar expression patterns.

### Analysis of the draft composite genome of KB-1 *Dhc*

Identification of sequences belonging to *D. mccartyi* strains within the assembled KB-1 metagenome sequence resulted in an initial draft genome containing 209 contigs. After PCR-based directed sequencing and fosmid clone sequencing, the final draft of the KB-1 *Dhc* genome consists of 32 contigs (Table S11 in File S1). Of these, 29 are relatively short in length (2,700–37,000 bp). The longest complete contig represents 1.31 Mb of a ∼1.4 Mb genome and encompasses the complete core region of sequenced *D. mccartyi* genomes. One 60 kb contig represents an alternative to the high plasticity region #1 (HPR1) of a *D. mccartyi* genome [22] with end regions that perfectly overlap the HPR1 flanking regions in the main genome scaffold. The two possible HPR1 regions are complete, while the structure of the HPR2 region(s) remains largely undefined. The complete draft scaffold has a GC content of 47.2% and is 1.76 Mb long; this is larger than the previously published *D. mccartyi* genomes ranging from 1.34–1.47 Mb [12], [13], [22], [45]. It is likely that not all of these smaller contigs belong to the same genome, and the presence of strain variation is contributing to the difficulty in closing the HPR2 region as that is the prime candidate region for genomic rearrangements in *D. mccartyi* [22]. The core genome region, thus, represents a chimeric assembly of two (or more) *D. mccartyi* strains within the KB-1 community. In this case, the level of strain variation was not sufficient to disrupt the assembly algorithms and is impossible to segregate without independent sequence data from at least one of the strains. Open reading frame calling and annotation resulted in a total of 1615 predicted genes (Table S12 in File S1), which were used to identify the KB-1 shotgun array sequences belonging to *D. mccartyi* from the total KB-1 community arrays. Included in the 1615 genes were 32 *rdh*A genes, a complement in line with the known gene complements of previously sequenced genomes (ranging from 11–36 *rdh*A per genome) [12], [13], [22], [45].

### Prediction of operon structures for *D. mccartyi* genomes

We predicted the operon structures of strain 195 genome and the draft composite genome of KB-1 *Dhc* with a published operon prediction algorithm [46]. This algorithm was chosen because of its improved prediction capability for a newly sequenced genome and ease of implementation as it does not require any experimental data [46]. Since operons are sets of multiple co-transcribed genes forming a single mRNA sequence [47], they encode proteins of similar metabolic or regulatory functions; hence, this information, together with co-expressed gene clusters, can be used to infer functions for hypothetical proteins and proteins with unknown functions [48]–[50]. Of the total 1589 and 1615 genes in the genome of strain 195 and in the contigs from KB-1 *Dhc*, 1251 (79%) and 984 (61%) were identified to be part of an operon (i.e., operonic) comprising 348 and 318 multigene operon pairs, respectively (Table S13 in File S1). Due to the low number (61%) of predicted operonic genes in KB-1 *Dhc*, we tested the prediction capability of the algorithm by applying it to two other publicly accessible and complete *D. mccartyi* genomes — strains CBDB1 and GT — that share high nucleotide similarity and gene synteny with KB-1 *Dhc* [51]. Strain CBDB1 contains 79% (1150 of 1457) operonic genes consisted of 333 multigene operon pairs, while strain GT has 295 such operon pairs comprising 78% (1119 of 1432) of genes in the genome (Table S13 in File S1). Our operon predictions for strains 195 and CBDB1 (79% for each) are comparable to the publicly available results for those genomes (71% and 76%) in the DOOR database [52] (Table S13 in File S1). Operon prediction result for the composite genome of KB-1 *Dhc* was lower because only a draft genome assembled from the KB-1 metagenome is available, and contig breaks can disrupt operons (see Materials and methods).

### Clustering and functional enrichment analyses of transcriptomic data

**Figure 5:**
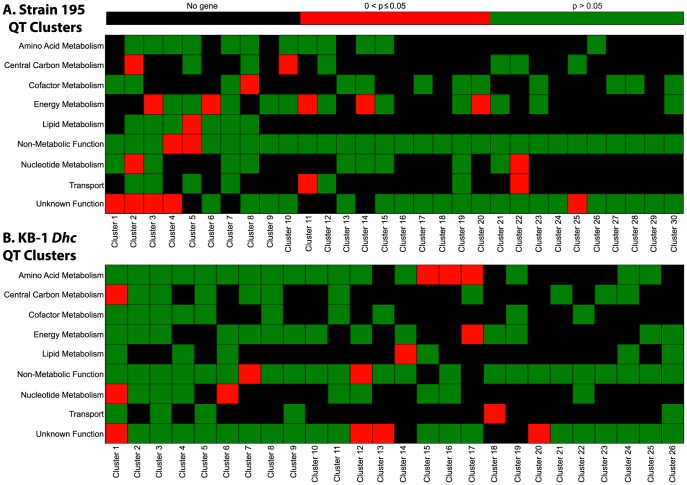
Functional enrichment analysis of QT clusters for (A) strain 195 and (B) KB-1 *Dhc* array data. Genes in each QT cluster were categorized according to the subsystems or functional categories of *D. mccartyi* metabolic model. Next, enrichment p-values were calculated using hypergeometric distribution for each QT cluster to identify which clusters were enriched with genes from a particular subsystem. This analysis identified 13 and 11 clusters of co-expressed genes for strain 195 and KB-1 *Dhc*, which were significantly overrepresented by genes from specific functional categories. Such functionally enriched clusters are shaded in red (p≤0.05) while black (No gene) indicates the absence of a gene from the corresponding subsystems, and green represents non-significant p-values (p>0.05) for the clusters.

**Figure 6:**
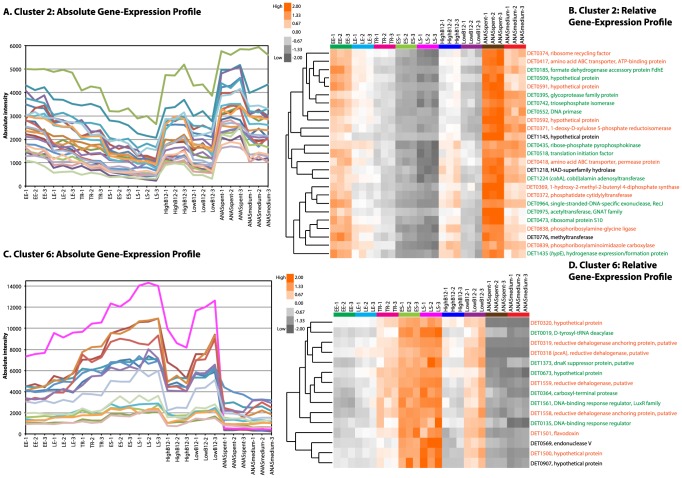
Analysis of two functionally enriched strain 195 QT clusters. Two functionally enriched and interesting QT clusters (clusters 2 and 6) of strain 195 transcriptomic data were further analyzed by the hierarchical clustering algorithm as represented by the dendrograms in (B) and (D). Absolute gene expression intensities of the clusters are plotted in (A) and (C), while relative or normalized gene expression intensities (see Materials and methods) are presented as heat maps in (B) and (D). The height of the dendrograms represents the similarity of gene transcription patterns and is measured by the Spearman's rank correlation coefficient (SCC). Genes whose names are in green or orange are part of an operon, but orange further indicates that multiple genes from the same operon are present in the cluster.

In addition to confirming the expression of sequenced genes in strain 195 and KB-1 *Dhc* genomes, we also analyzed the transcriptomic data for both organisms with the quality threshold (QT) clustering algorithm [53] to identify clusters of co-expressed or co-transcribed genes [49]. QT clustering is an unsupervised algorithm that ensures the quality of formed gene clusters by applying such quality thresholds as minimum cluster diameter and cluster size [53]. Using very stringent cut-offs of the algorithm (see Materials and methods), we obtained 30 QT clusters of 7–31 genes for strain 195 and 26 QT clusters of 7–35 genes for KB-1 *Dhc* (Tables S14 and S15 in File S1). In the *D. mccartyi* metabolic model [23], all metabolic genes were categorized in seven model subsystems (i.e., functional categories) based on their involvement in different metabolic pathways [23]. This information was used to identify and categorize metabolic genes belonging to each QT cluster (see Materials and methods, and Figures S1 and S2 in File S2). Also, hypothetical proteins and genes without any particular annotations were categorized as “unknown function”, while genes involved in regulation, DNA repair, replication, and recombination were classified as “non-metabolic function” in the QT clusters. Subsequently, functional enrichment analysis [54]–[56] (Figure 5) was performed for all QT clusters, and enrichment p-values were calculated using the hypergeometric distribution method [54]. We obtained 13 and 11 functionally enriched i.e., overrepresented (p<0.05) QT clusters for strain 195 (Figure 6A) and KB-1 *Dhc* (Figure 6B), respectively.

Functional enrichment analysis [54]–[56] was performed to obtain better insight into the contents of each co-expressed QT cluster. Although each gene cluster contains important information, functionally enriched clusters emphasize the presence of genes from a certain functional category is statistically significant, and potentially all genes in the cluster may be related to similar functions, or involved in similar metabolic pathways (see Tables S14 and S15 in File S1 for a list of all QT clusters and genes). These clusters are, therefore, useful in predicting and analyzing the functions of hypothetical proteins within them. Of the 13 and 11 functionally enriched clusters of strain 195 and KB-1 *Dhc*, some are enriched for more than one functional category (Figures 5A and 5B). This multiple enrichment situation indicates that genes belonging to the enriched categories are probably functionally related, or may be regulated by common regulators. Also, the clusters enriched for energy metabolism genes, such as hydrogenases, reductive dehalogenases, and proton translocating NADH-dehydrogenases are important for organohalide respiring *D. mccartyi*. Thus, further analysis of two such QT clusters of strain 195 (Figure 6) is described in the following sections, and summarized in Table 2 and Table S16 in File S1.

**Table 2:** Strain 195 genes identified in functionally enriched clusters and associated inferred annotations*

Locus Tag	Operon ID	Cluster No	Model Gene No	Primary Annotation	Revised Annotation in the Model	Suggested New Annotation from This Study	Subsystem
DET0509	106	2	113	hypothetical protein	putative bifunctional phosphoglucose/phosphomannose isomerase	Retain previous annotation	Central Carbon Metabolism
DET0742	160	2	192	triosephosphate isomerase	Retain previous annotation	Retain previous annotation	Central Carbon Metabolism
DET0369	84	2	57	1-hydroxy-2-methyl-2-(E)-butenyl 4-diphosphate synthase	Retain previous annotation	Retain previous annotation	Lipid Metabolism
DET0371	84	2	58	1-deoxy-D-xylulose 5-phosphate reductoisomerase	Retain previous annotation	Retain previous annotation	Lipid Metabolism
DET0372	84	2	59	phosphatidate cytidylyltransferase	Retain previous annotation	Retain previous annotation	Lipid Metabolism
DET0417	91	2	79	amino acid ABC transporter; ATP-binding protein	putative glutamine transporter	putative methionine transporter	Transport
DET0418	91	2	80	amino acid ABC transporter; permease protein	putative glutamine transporter	putative methionine transporter	Transport
DET0518	108	2	118	translation initiation factor, putative,	methylthioribose-1-phosphate isomerase	Retain previous annotation	Amino Acid Metabolism
DET0591	125	2		hypothetical protein	hypothetical protein	putative carbohydrate esterase	Central Carbon Metabolism
DET0592	125	2		hypothetical protein	hypothetical protein	putative Maltose 6-phosphate glucosidase	Central Carbon Metabolism
DET0318	71	6	19	reductive dehalogenase, putative	tetrachloroethene reductive dehalogenase	Retain previous annotation	Energy Metabolism
DET0319	71	6	446	reductive dehalogenase anchoring protein, putative	tetrachloroethene reductive dehalogenase anchoring protein	Retain previous annotation	Energy Metabolism
DET0320	71	6		hypothetical protein	hypothetical protein	putative transcriptional regulator/activator	Non-metabolic
DET1558	326	8	523	reductive dehalogenase anchoring protein, putative	Retain previous annotation	Retain previous annotation	Energy Metabolism
DET1559	326	8	425	reductive dehalogenase, putative	Retain previous annotation	Retain previous annotation	Energy Metabolism
DET1500	310	8		hypothetical protein	hypothetical protein	putative transcriptional regulator/activator	Non-metabolic
DET1501	310	8		flavodoxin	flavodoxin	Retain previous annotation	Energy Metabolism

*A more detailed version of this table is presented in Table S16 in File S1, where rationales for each reannotation are described briefly.

### Predicting functions for hypothetical proteins from the analysis of strain 195 QT cluster 2

Cluster 2 of strain 195 comprises 25 genes and is overrepresented by genes from central carbon metabolism, nucleotide metabolism, and of unknown function (Figure 5A). The absolute gene-expression profile (Figure 6A) shows that genes in this cluster have similar expression patterns with higher expression in “HighB12” and “ANASspent” conditions. However, the relative gene-expression profile (Figure 6B) indicates the genes were most highly and lowly transcribed in “ANASspent” and “LS” conditions, respectively. Since genes in this cluster are mostly growth related, as suggested by the enrichment of genes from central carbon metabolism and nucleotide metabolism categories, higher gene-transcription in those samples likely indicates a faster growth of strain 195. Also, the filter sterilized supernatant of ANAS culture (i.e., ANASspent) added growth medium probably had the highest nutrient content [28], [35] as compared to the rest of the conditions; hence, higher transcription of genes in the “ANASspent” condition (Figure 6B) was likely due to the favourable growth of strain 195. The lowest concentration of substrate and nutrient in the “LS” condition resulted in slow growth of strain 195 [27] and was possibly responsible for the lowest gene-transcription in this condition (Figure 6B). In the metabolic modeling study [23], the central metabolic genes (DET0509 and DET0742) of this cluster were suggested to be involved in glycolysis/gluconeogenesis and sugar metabolism to produce precursors for cell membrane biogenesis [57]–[59]. DET0509 (hypothetical protein) was annotated as a putative bifunctional phosphoglucose isomerase (EC: 5.3.1.8)/phosphomannose isomerase (EC: 5.3.1.9) during extensive curation of the *D. mccartyi* metabolic model [23] (Table 2 and Table S16 in File S1). Thus, its inclusion in a central carbon metabolism gene-enriched cluster further supports its proposed annotation. Similarly, two other operonic hypothetical proteins, DET0591 and DET0592 (Figure 6B and Table 2), of this cluster are probably involved in sugar or carbohydrate metabolism because they clustered closer to the central metabolic genes (DET0509 and DET0742) during hierarchical clustering (Figure 6B). Moreover, two other genes (DET0590: glyceraldehyde-3-phosphate dehydrogenase and DET0593: enolase) of this operon [58] are also involved in sugar metabolism [57]. In fact, DET0592 is 58% identical at the amino acid level to the biochemically characterized maltose-6-phosphate glucosidase (EC: 3.2.1.122) of *Fusobacterium mortiferum* [60] in SWISSPROT [61] and PDB [62]; hence, it was annotated as putative maltose-6-phosphate glucosidase involved in carbohydrate metabolism [57] (Table 2 and Table S16 in File S1).

The cluster also includes three putative lipid metabolism genes that are members of the same operon: DET0369, DET0371, and DET0372 (Figure 6B and Table 2). DET0369 (EC: 1.17.7.1) and DET0371 (EC:1.1.1.267) involve in isoprenoid biosynthesis using the non-mevalonate pathway [57], [63]–[65], while DET0372 (phosphatidate cytidylyltransferase, EC: 2.7.7.41) takes part in glycerophospholipid metabolism [57], [58], the main structural components of biological cell membranes [59] (Figure 6B and Table 2). Two operonic transporter genes (DET0417 and DET0418) were proposed to be putative L-glutamine transporters during the metabolic modeling study [23]; however, clustering of DET0418 closer to DET0518 (Figure 6B) suggests that both are probably methionine transporters. This is because the proposed annotation of DET0518 was a putative methylthioribose-1-phosphate isomerase (EC: 5.3.1.23), involved in methionine metabolism [57], [58], in the modeling study [23]. Intriguingly, the close hierarchical clustering of a putative methionine transporter (DET0418) with a gene involved in glycerophospholipid metabolism (DET0372) (Figure 6B and Table 2) suggests a potential relationship between amino acid transport and lipid metabolism during strain 195's growth because both are growth related. A recent isotope labelling study [21], indeed, showed that strain 195 incorporated methionine from the external medium during growth and dechlorination. Thus, QT clustering analysis of transcriptomic data, along with functional enrichment analysis and operon predictions, helped annotate hypothetical proteins, or propose new annotations for previously annotated genes of strain 195.

### Insight into *D. mccartyi* electron transport chain from the analysis of strain 195 QT cluster 6

Another important QT cluster of strain 195, overrepresented by genes involved in energy metabolism (Figure 5A), is cluster 6 comprising 15 genes. Absolute (Figure 6C) and relative (Figure 6D) gene expression profiles of this cluster showed high and low transcription of genes in” LS” and “ANASspent” conditions, respectively — a scenario opposite to the previously described QT cluster 2. This difference in relative gene expression profiles suggests that strain 195 needs to generate energy by reductive dechlorination to maintain cellular integrity [66]–[68] even though the cells are not growing in the “LS” condition. It also supports the notion of growth-decoupled reductive dechlorination by this bacterium [7], [13]. Genes in this cluster are mainly involved in energy metabolism, specifically genes present in the respiratory chain of strain 195, including two *rdh*A and two *rdh*B genes (DET0318, *pce*A, DET0319, DET1558, and DET1559) (Table 2 and Figure 6D). Interestingly, DET0318 — a biochemically characterized tetrachloroethene (PCE) *rdh*A (*pce*A) gene [18] — was not transcribed in “ANASspent” and “ANASmedium” conditions though it was the most highly transcribed gene during the growth of strain 195 in its own medium (Figure 6C). ANAS cultures were not reported to degrade PCE [16], [35], and the supernatant, as well as the growth medium of ANAS might contain nutrients that possibly inhibited the *pce*A gene expression.

The cluster also contains a putative flavodoxin gene (DET1501) that is 33% identical at the amino acid level with the biochemically characterized flavodoxin from *Desulfovibrio vulgaris* strain Hildenborough [69] in SWISSPROT and PDB (Table 2 and Figure 6D). Flavodoxins are small electron transfer proteins containing a single flavin mononucleotide (FMN) molecule that usually participates in low potential redox reactions [70], [71]. Thus, the presence of a putative flavodoxin (DET1501) with *rdh* genes in a co-expressed and energy metabolism gene-enriched QT cluster indicates its potential involvement in the reductive dechlorination process, as well as in the respiratory chain of strain 195 (Figure 6D, Table 2, and Table S16 in File S1). This hypothesis is further corroborated by the fact that a low potential electron donor is required to continue reductive dechlorination by *D. mccartyi* [1], [11], [72]. Recently, a flavin mediated “electron bifurcation” mechanism has been reported for anaerobic microorganisms [73], [74], in which an endergonic reaction is driven by the energy from a simultaneously occurring exergonic reaction. The mechanism of *D. mccartyi* electron transport chain (ETC) is still unknown; however, probable involvement of a flavodoxin, together with reductive dehalogenases in the ETC suggests the possibility of electron bifurcation during the reductive dechlorination process. Surprisingly, no flavodoxin gene was found in *D. mccartyi* strain VS which warrants further investigation. Also, the inclusion of DET0320 and DET1500 — two putative transcriptional regulators due to their homology (46% amino acid sequence identity with *E. coli* K12) in SWISSPROT, IMG, PDB, and EBI InterProScan [75] databases — in this cluster suggests their likely involvement in regulating energy conservation processes and reductive dehalogenation, as has been suggested previously [12], [13] (Table 2 and Table S16 in File S1). Clustering of similar energy metabolism genes was also observed for KB-1 *Dhc* transcriptomic data (Table S16 in File S1).

Although gene expression microarrays are genome-wide high throughput experimental studies cataloguing the global transcriptional changes of an organism, they cannot provide deterministic information such as the activity of genes and enzymes, or their involvement in specific metabolic processes. Hence, this information alone lacks the capability of unraveling and depicting the activity of metabolic genes, as well as the metabolism of an organism. However, if transcriptomic data can be analyzed together with detailed metabolic information such as a pan-genome-scale metabolic reconstruction as discussed in this study, they can provide useful insights about the function of metabolic genes, as well as hypothetical proteins. Such integrated analysis can also be instrumental in shedding light on poorly understood physiological processes of difficult to culture organisms like *D. mccartyi*. That being said, the transcriptomic experiments and data analyzed in this study were not designed specifically to capture the changes in expression pattern of metabolic genes; for instance, *D. mccartyi* were either growing or not-growing in all experimental conditions, and no specific metabolic perturbations such as the lack of an essential nutrient or vitamin were imposed on them during their growth. Moreover, absolute expression intensities, rather than differential gene-expression analysis, of array data were used in this study due to the variability of array design methods and array data sources. Hence, future microarray experiments designed to perturb and catalogue metabolic changes in *D. mccartyi* will be useful for advancing our fundamental understanding about the physiology and metabolism of these environmentally important yet difficult to culture microbes.

## Conclusions

Due to the lack of a genetic system and associated challenges of growing pure isolates of *D. mccartyi* in defined mineral media, detailed biochemical studies concerning their physiology and metabolism are limited. This study analyzed and visualized curated transcriptomic data for strain 195 and *D. mccartyi* strains in KB-1 (KB-1 *Dhc*) from various experiments while leveraging our previously developed *D. mccartyi* metabolic network and model. Using the transcriptomic data, as well as the proteomic data from previous studies, we confirmed the presence of the majority of hypothetical proteins and metabolic genes in strain 195 and KB-1 *Dhc* genomes. We identified a number of high quality clusters for both data sets that provided improved understanding of the genes (such as flavodoxin and *rdh*s) involved in the yet unknown mechanism of the energy conserving respiratory chain of these organisms. Clustering and functional enrichment analyses of the transcriptomic data highlighted that lipid metabolism, more specifically, cell membrane biogenesis and the function of transporters were very important for *D. mccartyi*. Operon analysis, as well as the quality threshold clustering of transcriptomic data, provided additional confidence in prior reannotations, or new function predictions for a number of hypothetical proteins. Since hypothetical proteins constitute a major portion of any sequenced genome, predicting function is a significant challenge, and all relevant clues are welcome. Also, predicted annotations for the hypothetical proteins can serve as a guide in designing future biochemical experiments for functional characterization of these genes. The techniques and analysis tools implemented in this study can be used for solving such problems in other systems. Finally, this study clearly shows that the integrated analysis of high-throughput transcriptomic data with the pan-genome-scale reconstructed metabolic network of *D. mccartyi* can advance our knowledge on the fundamental characteristics of the physiology and metabolism of these specialized anaerobes. This enhanced knowledge of metabolism, in turn, will be beneficial for the optimal use of these bacteria in elucidating global halogen cycles and developing effective strategies for the bioremediation of chlorinated pollutant contaminated sites around the world.

## Materials and Methods

### The draft composite genome of *D. mccartyi* strains in KB-1 (KB-1 *Dhc*)

The completed KB-1 metagenome (publically available in JGI-IMG and at genome.jgi-psf.org/aqukb/aqukb.download.ftp.html) was compared to five publicly available sequenced *D. mccartyi* genomes (strains 195, VS, BAV1, CBDB1, and GT) using “nucmer” program from the MUMmer package [76]. The contigs with identified homology to these reference genomes were parsed out and utilized for the initial attempts at *D. mccartyi* genome closure. Once RITA [77] classifications (http://kiwi.cs.dal.ca/Software/RITA) were available for the KB-1 contigs and singletons, additional *D. mccartyi* sequences were added to the draft genome. The initial *D. mccartyi* contigs were mapped to strain CBDB1 reference genome using Mauve version 2.3.0 [78]. The Mauve alignment was used to determine an initial expected order and orientation of *D. mccartyi* contigs in KB-1. A preliminary round of primers was designed for gap closure using Projector 2's [79] web interface (http://bioinformatics.biol.rug.nl/websoftware/projector2/projector2_start.php). PCR amplifications to close gaps in *D. mccartyi* scaffold contained 0.5 mM of each primer, 1× NEB *Taq* polymerase reaction buffer, 0.25 mM dNTPs, and 1–2 U *Taq* polymerase (NEB). Reactions were conducted at an annealing temperature of 54°C, an extension time commensurate with the predicted gap size (30 s – 4 min), and a total of 35 cycles of amplification. Template DNA for the reactions was either 0.1 µL of KB-1 genomic DNA from the original metagenome DNA sample, or a small aliquot of a fosmid library of frozen clone stock.

After PCR-based gap closing methods had been exhausted, all metagenome reads were mapped to the *D. mccartyi* scaffold using Geneious read mapping tool [80]. Fosmids whose mate pair reads were located on different contigs were identified, picked from the frozen library plate stocks, and inoculated into 5 mL overnight LB media cultures with 50 µg/mL chloramphenicol. An induction culture containing 0.3 mL of the overnight culture, 1.2 mL of LB, 50 µg/mL of chloramphenicol, and 1.5 µL of Epicenter Copy Control Induction Solution was grown with shaking for 5 hours. Induced cultures were centrifuged for 15 min at 8000×g, and fosmids extracted using the Qiagen plasmid midi-prep kit with the modified protocol for large insert or fosmid vectors. The purified fosmid DNA samples were pooled if they spanned the same gap on the genome, and the resulting 8 samples were barcoded and sequenced on a Roche 454 machine. Barcoded samples were assembled using Newbler (Roche), and the assembled contigs were combined with the existing *D. mccartyi* scaffold using minimus (http://amos.sourceforge.net).

### Identification of *D. mccartyi* genes from the KB-1 community shotgun microarray data

Pre-processed and normalized transcriptomic data for the KB-1 community were collected from a shotgun microarray study of 33 KB-1 samples [30], [31] and used for principal component analysis. Details of array construction methods, experimental conditions, and array data normalization techniques were described elsewhere [30], [31]. Although the KB-1 mixed microbial community mainly comprises dechlorinators, methanogens, acetogens, and fermenters [31], [37], [81], [82], *D. mccartyi* are the dominant members that detoxify toxic chlorinated solvents [31], [37], [81], [82]. In addition, only *D. mccartyi*-specific array data can be integrated with the pan-genome-scale metabolic reconstruction and model [23]; hence, only those genes and the corresponding array data were analyzed in this study. The data were extracted from KB-1 arrays and nucleotide sequences following a simple workflow (Figure S1 in File S2). First, all array sequences were aligned against the non-redundant nucleotide database (“nt”) from NCBI (http://www.ncbi.nlm.nih.gov/nuccore) with BLAST (blastn) [83] for identifying their species level identity. Sequences that matched to a database *D. mccartyi* genome as the best hit with >85% identity at the nucleotide level were chosen as *D. mccartyi* genes. Next, all array sequences were compared to the NCBI non-redundant protein database (“nr”) (http://www.ncbi.nlm.nih.gov/protein) with BLAST (blastx) [83] for identifying their annotations. Since *D. mccartyi* genomes are very similar [12], [13], [23], [51], only sequences that matched to the database *D. mccartyi* genes with >95% identity at the amino acid level were retained for subsequent analyses. Finally, KB-1 array nucleotide sequences were compared to the draft composite genome of KB-1 *Dhc* as constructed from the KB-1 metagenome [44], [84]. Afterwards, results from all three analyses were compared, and only consensus array sequences and corresponding intensity data were selected as the KB-1 *Dhc* array data (Figure S1 in File S2). Out of a total of 26,186 sequences from the KB-1 community shotgun arrays, 1,162 consensus sequences were identified as *D. mccartyi*. Subsequently, the data were analyzed with QT clustering algorithm [53], followed by mapping to the *D. mccartyi* metabolic network [23] for conducting functional enrichment analysis of the clusters [54]–[56] (Figure S1 in File S2).

### Processing of *D. mccartyi* strain 195 microarray data

Pre-processed and normalized transcriptomic data for *D. mccartyi* strain 195 was obtained from published literature [27], [28] and NCBI GEO database (http://www.ncbi.nlm.nih.gov/geo/). In total, microarray data for 9 experimental conditions and 27 samples were analyzed, where each condition comprised 3 parallel biological replicates. Of the total 1,579 array sequences, 1,560 non-duplicate sequences and corresponding array data from 27 samples were further analyzed following a workflow (Figure S2 in File S2). After PCA, array data for all samples were mapped to the *D. mccartyi* metabolic network for identifying metabolic genes followed by clustering of genes with the QT clustering algorithm [53]. Then functional enrichment analysis was performed through calculation of enrichment p-values for metabolic genes in each cluster with hypergeometric distribution method (Figure S2 in File S2).

### Operon predictions for *D. mccartyi* genomes

Operon predictions for both KB-1 *Dhc* and strain 195 were performed using the procedure described in Bergman et al. [46]. As per the procedure, we randomly chose 27 diverse bacterial genomes (Table S17 in File S1) from different branches of the bacterial phylogeny for constructing the barcode. The barcode was generated by identifying homologs of strain 195 and KB-1 *Dhc* from the chosen bacterial genomes. Subsequently, intergenic distance for each gene was calculated from the positional information of genes in the genome. Intergenic distance and strand location, as well as the barcode information was then used for calculating posterior probabilities of genes to be considered as operonic or not operonic. If the probability value of a gene was ≥0.5, it was assigned as an operonic gene; otherwise, genes were not considered to be operonic for lower probability values (Table S13 in File S1). A similar procedure was followed for identifying operon structures of strains CBDB1 and GT (Table S13 in File S1).

### Microarray data analysis and visualization

QT clustering analysis and heat map visualization of transcriptomic data were conducted with MeV: MultiExperiment Viewer [85] — an open-source software for analyzing and visualizing microarray gene-expression data. First, the array data were mapped to the reconstructed *D. mccartyi* metabolic network for identifying metabolic genes and classifying them according to the network subsystems. Next, QT clustering algorithm [53] and Spearman's rank correlation coefficient as the distance metric [86] were used for clustering the transcriptomic data. The number of clusters generated by QT clustering depends on two parameters: cluster diameter and minimum cluster size; thus, threshold for a cluster diameter and minimum cluster size was chosen as 0.06 and 7 for obtaining very stringent QT clusters. Theses stringent cut offs also ensured that co-expressed or co-transcribed clusters formed were not very large and potentially be more meaningful. Using both subsystem and clustering information, hypergeometric p-values were calculated for each QT cluster to identify functionally enriched i.e., overrepresented (p≤0.05) clusters [54]–[56]. Subsequently, hierarchical clustering [87] was used for further analysis of some functionally enriched interesting QT clusters. Absolute intensity values were used for representing if a gene was transcribed (“on”) or not transcribed (“off”) in heat maps. The frequency distribution of intensity values (Figures S3 and S4 in File S2) showed that the majority of strain 195 and KB-1 *Dhc* genes were transcribed above intensity values of 800 and 100, respectively. Hence, we set the threshold intensity of 800 (<800 =  “off”, >800 =  “on”) for strain 195 data and 100 (<100 =  “off”, >100 =  “on”) for KB-1 *Dhc* arrays to represent as heat maps. Relative or normalized gene expression intensities were calculated using the formula: normalized intensity value  =  [(absolute intensity value) – mean of absolute intensity values in a row)]/[standard deviation of absolute intensity values in a row]. Because each row in the array data matrix contained data for a gene across different conditions, normalized intensities showed the highest and lowest transcription of any gene across all samples. Principal component analysis (PCA) of the array data was performed using the “princomp” function from the statistics toolbox in MATLAB (The Mathworks Inc.). The function performs PCA on the transpose of an m X n data matrix, where the rows are representing genes and the columns are samples or experimental conditions, and the function returns principal component coefficients.

## Supporting Information

File S1 **This excel file contains 17 supplemental tables:** Table S1. Proteomic and Transcriptomic Evidence for All Genes in Strain 195 Genome; Table S2. Proteomic and Transcriptomic Evidence for All Genes in KB-1 *Dhc* Genome; Table S3. Strain 195 Hypothetical Proteins Transcribed or “On” (≥800) in All Samples; Table S4. Strain 195 Hypothetical Proteins Not Transcribed or Not “On” (<800) in All Samples; Table S5. KB-1 *Dhc* Hypothetical Proteins Transcribed or “On” (≥100) in All Samples; Table S6. KB-1 *Dhc* Hypothetical Proteins Not Transcribed or Not “On” (<100) in All Samples; Table S7. Proteomic and Transcriptomic Evidence for Strain 195 Metabolic Genes; Table S8. Proteomic and Transcriptomic Evidence for KB-1 *Dhc* Metabolic Genes; Table S9. Expression of *rdh*A Genes of Strain 195; Table S10. Expression of *rdh*A Genes of KB-1 *Dhc*; Table S11. Contig Sequences of KB-1 *Dhc* Draft Genome; Table S12. Protein Sequences of KB-1 *Dhc* Draft Genome; Table S13. Operon Prediction Results for *Dehalococcoides mccartyi* Genomes; Table S14. Quality Threshold (QT) Clusters of Strain 195 Transcriptomic Data; Table S15. Quality Threshold (QT) Clusters of KB-1 *Dhc* Transcriptomic Data; Table S16. Selection of Genes Identified in Functionally Enriched Significant Clusters and Associated Inferred Annotations; and Table S17. List of Genomes Used for Operon Prediction.
(XLSX)

File S2 **This PDF file contains 4 supplemental figures:** Figure S1. Workflow for Analyzing Pre-Processed KB-1 Microarray Data; Figure S2. Workflow for Analyzing Pre-Processed Strain 195 Microarray Data; Figure S3. Distribution of Strain 195 Gene Expression Intensities for 27 Samples; and Figure S4. Distribution of KB-1 *Dhc* Gene Expression Intensities for 33 Samples. These figures are generated for explaining some methods and parameters used in the main text.
(PDF)
