# Supplementary material for: New Insights into Dehalococcoides mccartyi Metabolism from a Reconstructed Metabolic Network-Based Systems-Level Analysis of D. mccartyi Transcriptomes
Source: PLoS One. 2014 Apr 14;9(4):e94808. doi: 10.1371/journal.pone.0094808 (PMC3986231; doi:10.1371/journal.pone.0094808)
Supplement: File S2 — This PDF file contains 4 supplemental figures: Figure S1. Workflow for Analyzing Pre-Processed KB-1 Microarray Data; Figure S2. Workflow for Analyzing Pre-Processed Strain 195 Microarray Data; Figure S3. Distribution of Strain 195 Gene Expression Intensities for 27 Samples; and Figure S4. Distribution of KB-1 Dhc Gene Expression Intensities for 33 Samples. These figures are generated for explaining some methods and parameters used in the main text. (PDF) [file pone.0094808.s002.pdf]

File S2

New Insights into *Dehalococcoides mccartyi* Metabolism from a Reconstructed Metabolic Network-Based Systems-Level Analysis of *D. mccartyi* Transcriptomes

M. Ahsanul Islam, Alison S. Waller, Laura A. Hug, Nicholas J. Provart, Elizabeth A. Edwards, and Radhakrishnan Mahadevan*

*Corresponding author

ahsan.islam@utoronto.ca

alison.waller@embl.de

laura.hug@berkeley.edu

nicholas.provart@utoronto.ca

elizabeth.edwards@utoronto.ca

krishna.mahadevan@utoronto.ca

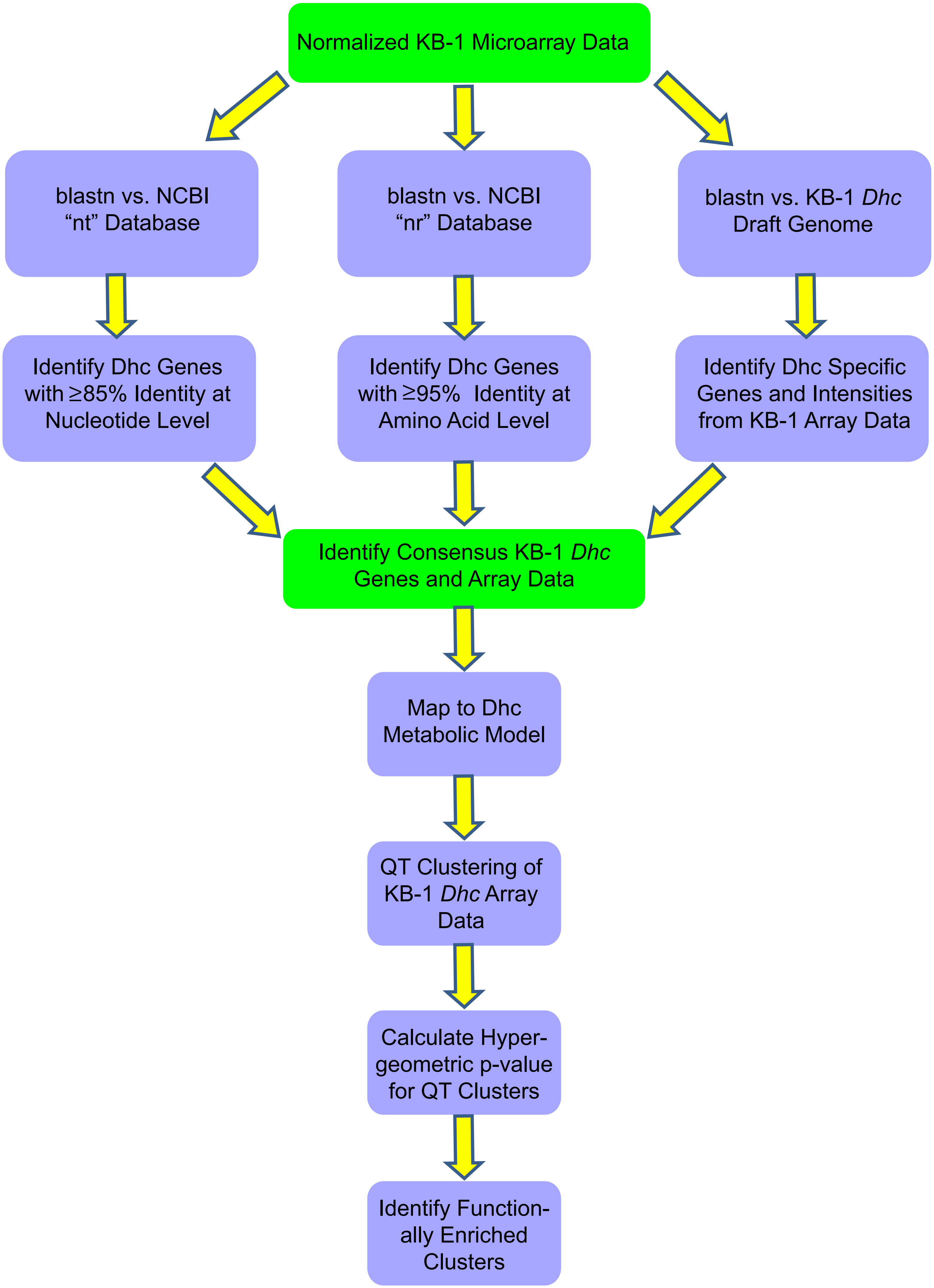

Figure S1. Workflow for Analyzing Pre-Processed KB-1 Microarray Data

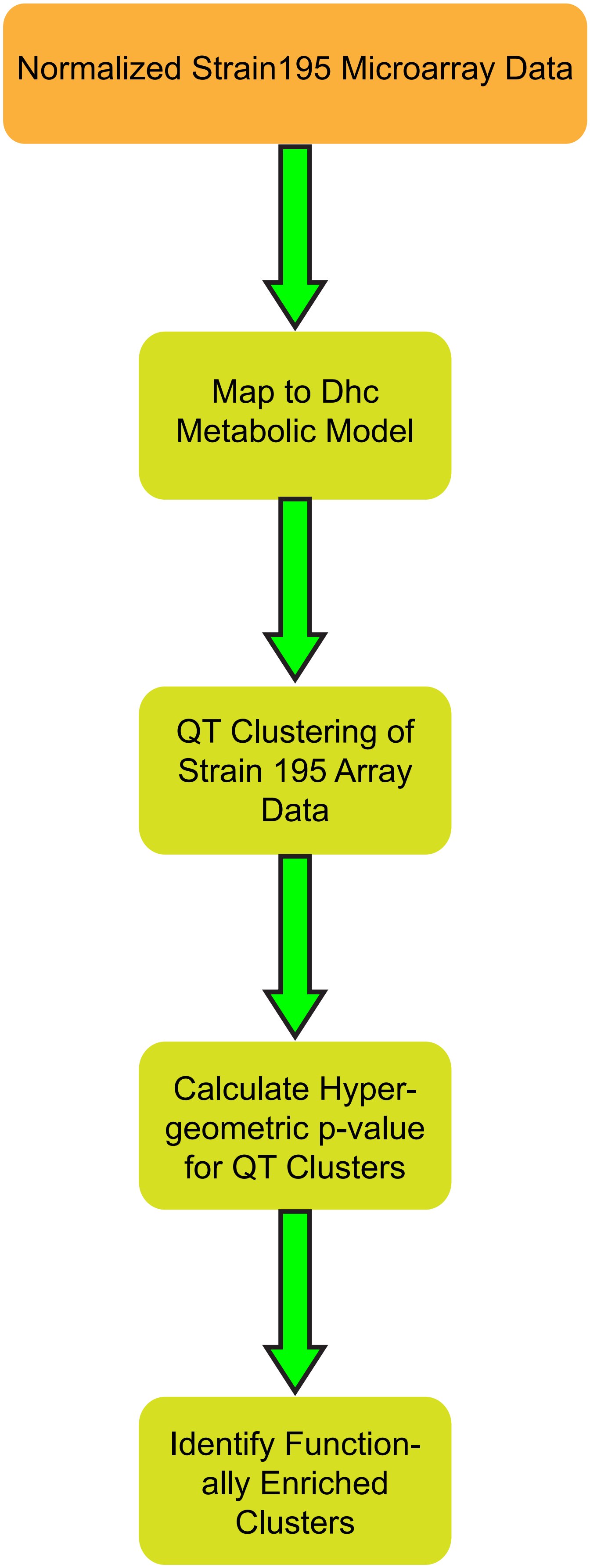

Figure S2. Workflow for Analyzing Pre-Processed Strain 195 Microarray Data

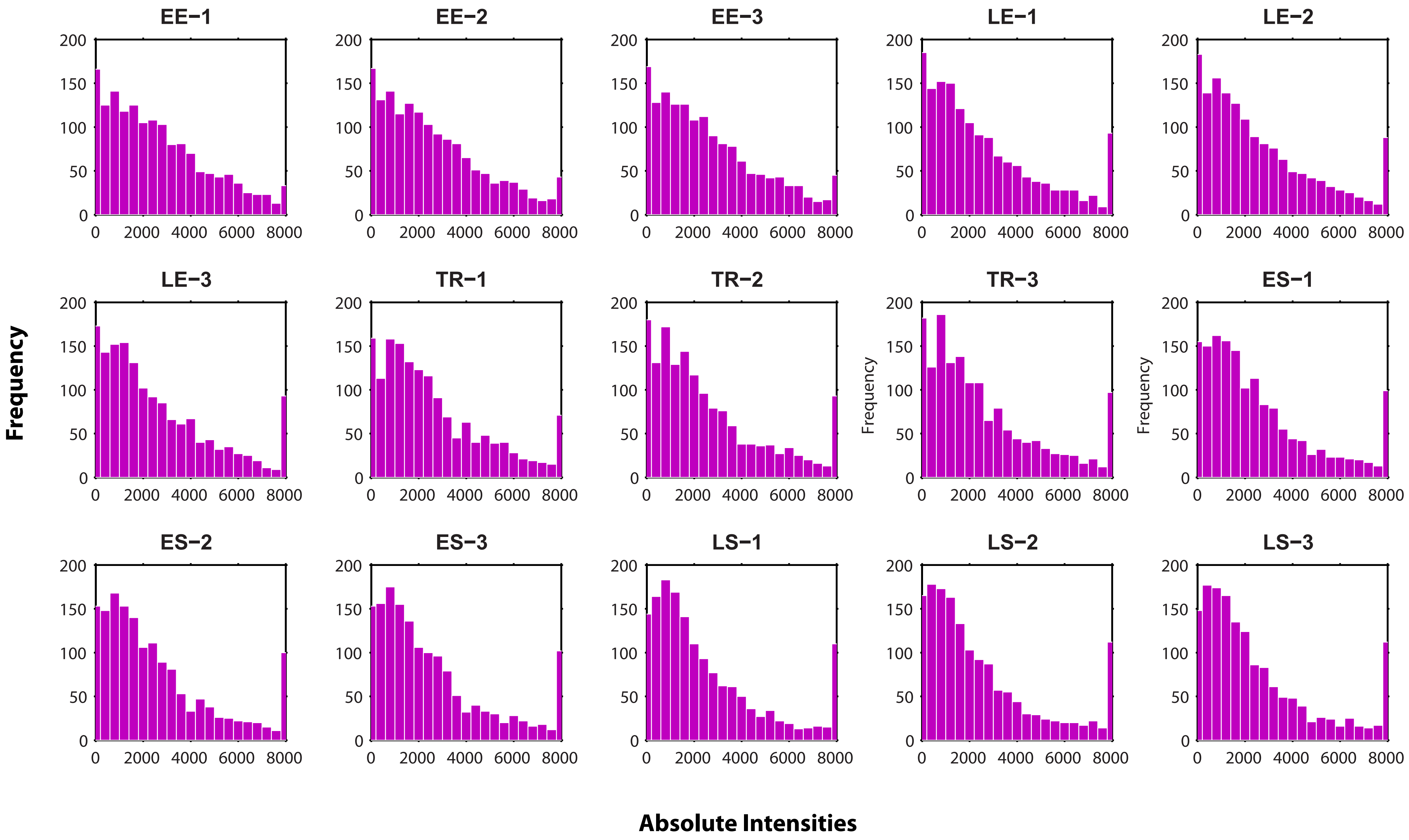

Figure S3. Distribution of Strain 195 Gene Expression Intensities for 27 Samples

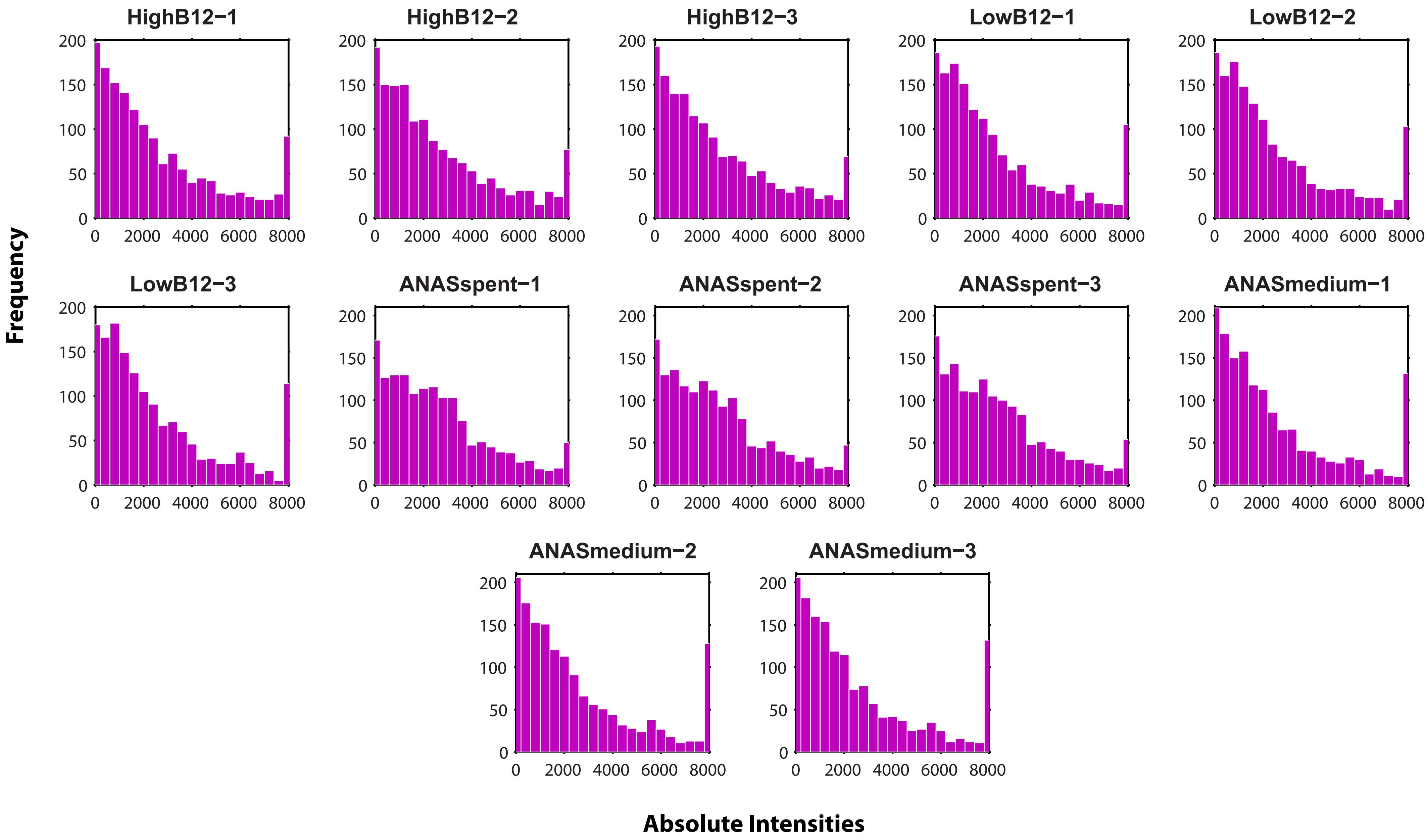

Figure S3. Distribution of Strain 195 Gene Expression Intensities for 27 Samples (continued from previous page)

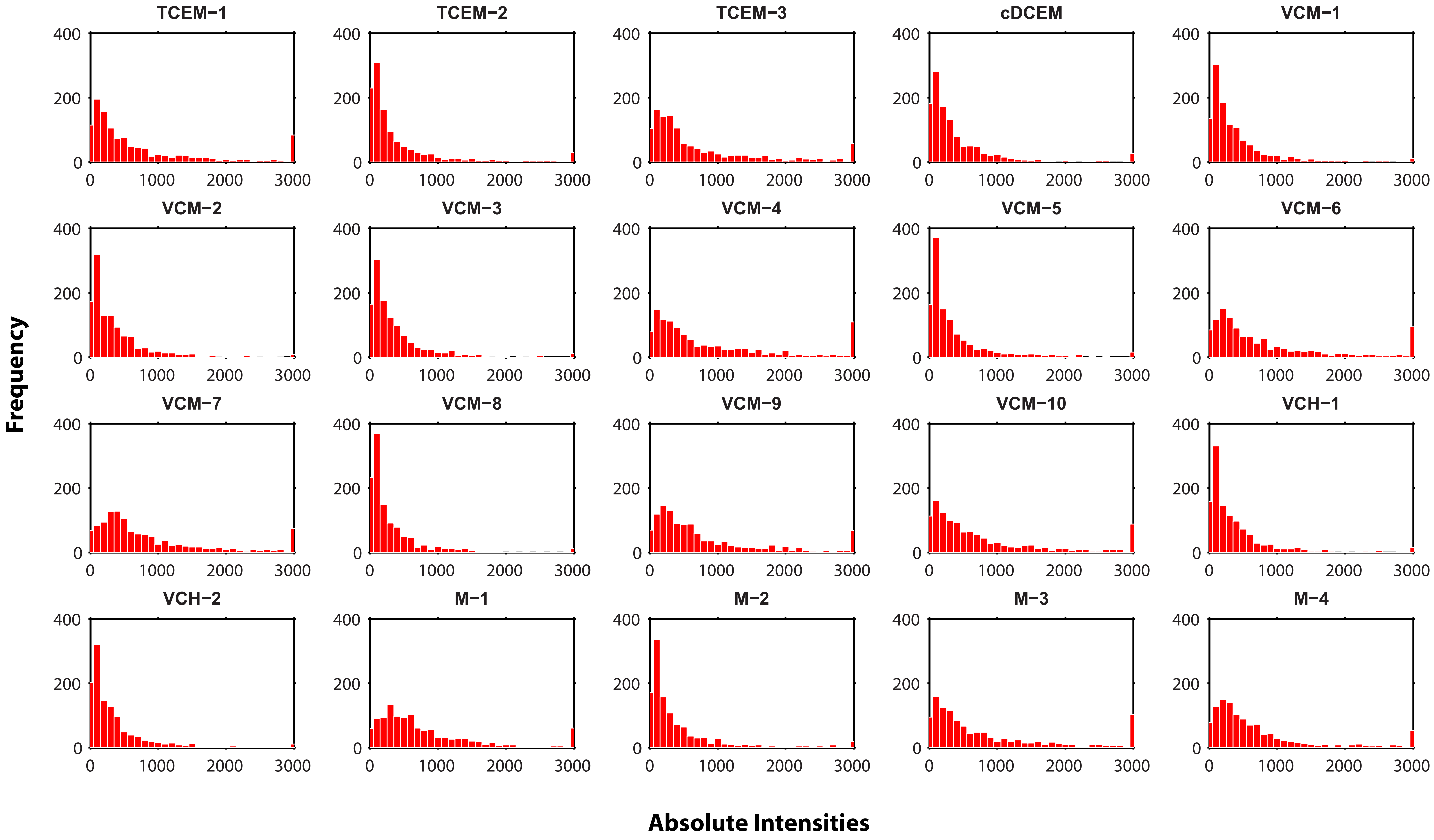

Figure S4. Distribution of KB-1 *Dhc* Gene Expression Intensities for 33 Samples

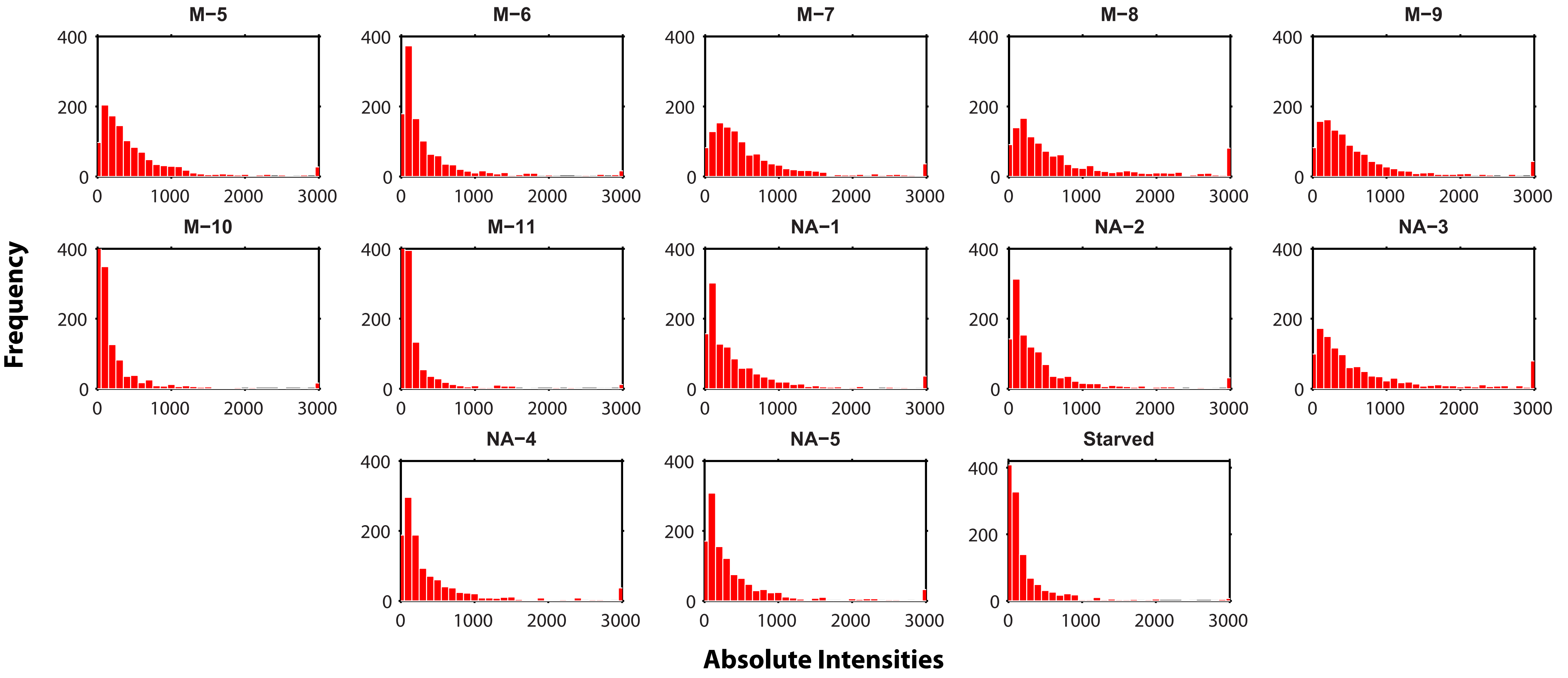

Figure S4. Distribution of KB-1 *Dhc* Gene Expression Intensities for 33 Samples (continued from previous page)
